# Using α radiation to boost cancer immunity?

**DOI:** 10.4161/21624011.2014.954925

**Published:** 2014-12-06

**Authors:** Jean-Baptiste Gorin, Yannick Guilloux, Alfred Morgenstern, Michel Chérel, François Davodeau, Joëlle Gaschet

**Affiliations:** 1 INSERM UMR 892 - CRCNA; Nantes, France; 2 University of Nantes; Nantes, France; 3 CNRS UMR 6299; Nantes, France; 4 Institut de Cancérologie de l’Ouest; Saint-Herblain, France; 5 Institute for Transuranium Elements; Karlsruhe, Germany

**Keywords:** abscopal effect, α emitters, cancer, immunogenic cell death, ionizing radiation, RIT, ^213^Bi, bismuth-213, DAMPs, danger associated molecular patterns, DC, dendritic cells, HMGB1, high mobility group box 1, Hsp70, 70 kilodalton heat shock protein, ^224^Ra, radium-224, RIT, radioimmunotherapy

## Abstract

Radioimmunotherapy aims to deliver radiation directly to cancer cells by means of a tumor specific vector coupled to a radionuclide. Alpha radionuclides are very potent agents to treat disseminated cancer and metastasis. We have demonstrated that α radiation can also induce immunogenic cell death, reinforcing interest in their clinical development.

Radiotherapy is one of the most effective tools in the battle against cancer and is now integrated in the treatment of more than half of cancer patients. Ionizing radiation is commonly used due to its tumoricidal capacity resulting from direct cytotoxicity potentially associated with bystander effects. Many radiotherapy modalities now exist, and constant progress in the development of new technologies has made it possible to deliver high doses of radiation to tumor sites with little toxicity to surrounding tissues.

Our group is particularly interested in the development of radioimmunotherapy (RIT), which is one of the most promising modalities for the treatment of disseminated cancers. This technique uses vectors capable of specifically targeting tumor cells once injected systemically or locally to deliver a radioactive load. One of the latest developments of this approach is the use of α particle emitters, which are highly energetic with a short path length in tissues, making them very attractive for the treatment of small clusters of tumor cells. Additionally, radiobiological effects associated to α radiation are less sensitive to dose rate, hypoxia, and cell cycle distribution than are β particles, γ rays or X-rays.^1^ These beneficial attributes led to the initiation of several clinical trials encompassing a variety of different malignancies.^2^ Furthermore, our team recently established proof-of-concept for the potential use of RIT, finding that ^213^Bi, an α particle emitter, solicits immunologic responses in a mouse model of multiple myeloma.^3^ Altogether, the data from different preclinical and clinical trials using α particles show great promise for the treatment of disseminated cancers.

Nevertheless, it has become increasingly clear that irradiation does not only act through direct cytotoxicity, as accumulating evidence shows that external treatment with γ and X rays can have a beneficial effect at distance from the field of irradiation, a phenomenon observed in both animal models and in the clinic.^4^ This phenomenon, known as the abscopal effect, may be triggered through immunogenic cell death of malignant cells accompanied by the release of danger associated molecular patterns (DAMPs) and the induction of an inflammatory context prone to attract and activate immune cells such as dendritic cells (DCs). Activated DCs can in turn cross-present antigens from the irradiated tumor cells to stimulate a specific T-cell responses against malignant cells.^5,6^

These observations prompted us to investigate the immunological potential of α particles. We therefore studied the capability of ^213^Bi in stimulating antitumor immunity against the murine adenocarcinoma MC-38. In order to avoid any bias due to the irradiation of the tumor microenvironment, and to focus only on the effect of ^213^Bi on tumor cells, we chose to irradiate MC-38 cells *ex vivo* and inject them in immunocompetent C57Bl/6 mice in a vaccination approach.^7^

We found that vaccination with ^213^Bi irradiated tumor cells protects immunocompetent hosts against further tumor challenge with the same tumor. Indeed, 88% of the animals vaccinated in one flank survived to the injection of live MC-38 tumor cells in the opposite flank, compared to 16% of animals in the control group. Strikingly, this protection was observed in all the surviving animals subjected to a second challenge with live MC-38 cells 2 months after vaccination. The response was Tcell mediated as demonstrated by the presence of specific cytotoxic T cells and by the lack of protection in nude mice. These results prove that irradiation of cancer cells with ^213^Bi can lead to the neutralization of other tumor cells in a distant site, even long after the vaccination. We also showed that ^213^Bi irradiation of MC-38 cells *in vitro* induced the release of DAMPs such as heat shock protein 70 (Hsp70) and high mobility group box 1 (HMGB1) and that the conditioned media from irradiated cells was able to trigger activation of DCs as evinced by morphological changes as well as by the upregulation of costimulatory molecules (i.e., CD40 and CD86). In summary, we found that ^213^Bi fits the immunogenic cell death model proposed by Kroemer's group and that treatment with α particles can be associated with specific, systemic, and durable antitumor responses, which could be highly beneficial for anticancer therapy (**Fig. 1**).

**Figure 1.**
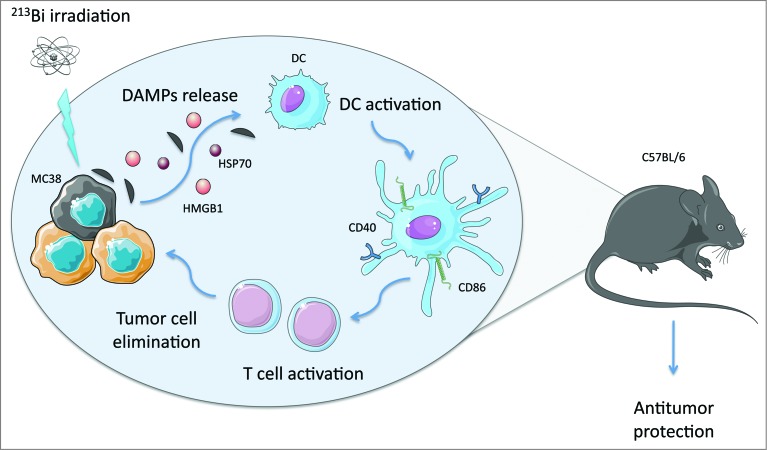
The radionuclide ^213^Bi induces immunogenic cell death that fosters effective antitumor immune protection *in vivo*. ^213^Bi irradiation of MC-38 adenocarcinoma cells causes them to release danger associated molecular proteins (DAMPs) such as heat shock protein 70 (HSP70) and high mobility group box 1 (HMGB1), thus activating dendritic cells (DCs). Activated DCs express costimulatory molecules that allow the activation of cytotoxic T cells specific for tumor antigens. T cells can then migrate in the body and eliminate remaining tumor cells at distant sites.

In support of our data, a recent paper from Keisari *et al.* showed that brachytherapy with α radiation in solid tumors could trigger an immune response against metastasis and augment memory immunity against tumor cells.^8^ They used ^224^Ra wires to treat subcutaneous breast or colon carcinoma and showed that α brachytherapy increased tumor protection against tumor rechallenge compared to surgical treatment. They also found that it reduced lung metastasis in the breast carcinoma model and that the efficacy of the treatment could be improved when associated with the immunostimulant CpG.

Altogether, these results strengthen the idea that ionizing radiation, in general, should not be employed exclusively for its cytocidal action. On the contrary, the immune aspect of radiation should be consistently taken into consideration and the development of newly envisioned radiotherapeutic schemes should be tested in immunocompetent hosts to address their immunogenic potential. Radiation-induced immunity also brings new and exciting perspectives into the anticancer fray for the use of α particle radiotherapy in combination with immunotherapeutic approaches. We are, indeed, convinced that the future of radiotherapy lies in conjunction with immunotherapy and we are confident that the years to come will see great progress in this direction.

## Disclosure of Potential Conflicts of Interest

No potential conflicts of interest were disclosed.
